# Two people, one graph: the effect of rotated viewpoints on accessibility of data visualizations

**DOI:** 10.1186/s41235-021-00297-y

**Published:** 2021-04-13

**Authors:** Tjark Müller, Friedrich W. Hesse, Hauke S. Meyerhoff

**Affiliations:** 1 Eberhardt-Karls University Tübingen, Tübingen, Germany; 2 FernUniversität Hagen, Hagen, Germany; 3 Leibniz-Institut Für Wissensmedien, Schleichstr. 6, 72076 Tübingen, Germany

**Keywords:** Mental rotation, Object recognition, Rotated views, Data visualization

## Abstract

In co-located, multi-user settings such as multi-touch tables, user interfaces need to be accessible from multiple viewpoints. In this project, we investigated how this goal can be achieved for depictions of data in bar graphs. We designed a laboratory task in which participants answered simple questions based on information depicted in bar graphs presented from differently rotated points of view. As the dependent variable, we measured differences in response onsets relative to the standard viewpoint (i.e., upright graphs). In Experiment 1, we manipulated graph and label orientation independently of each other. We observed that rotations of the labels rather than rotations of the graph itself pose a challenge for accessing depicted information from rotated viewpoints. In Experiment 2, we studied whether replacing word labels with pictographs could overcome the detrimental effects of rotated labels. Rotated pictographs were less detrimental than rotated word labels, but performance was still worse than in the unrotated baseline condition. In Experiment 3, we studied whether color coding could overcome the detrimental effects of rotated labels. Indeed, for multicolored labels, the detrimental effect of label rotation was in the negligible range. We discuss the implications of our findings for the underlying psychological theory as well as for the design of depicted statistical information in multi-user settings.

## Significance statement

Modern technology such as interactive multi-touch tables allows multiple users to access the same depicted information simultaneously. A central challenge with this technology is, however, that multiple users access the same information from different viewpoints. Consequently, depicted information is rotated from an upright orientation for all but one of the users. For effective group collaborations, however, displayed information should be accessible for all users, independent of their position at the table. In this project, we investigated this challenge for frequency information depicted in bar graphs. Our results showed that rotated views on bar labels rather than rotated views on the bars increased the difficulty of accessing depicted information. Further, we probed whether modifications of the standard bar labels could reduce such detrimental effects. Replacing word labels with pictographs reduced but did not eliminate the detrimental effects of rotated views. Color coding, however, effectively reduced the detrimental effects of rotated views to the negligible range. We therefore recommend color coding for depicting information in bar graphs in multi-user settings.

## Introduction

Imagine you are sitting opposite to someone who has spread out a newspaper across the table. One of the articles captures your interest, and despite the inverted orientation, you will be able to read the teaser of this article. Nevertheless, you might be slower and maybe less accurate than usual because the upside-down view on the article requires additional mental processes for recognizing and understanding the written text as well as the depictions (Hayward & Williams, 2000; Kolers, 1968). A similar situation arises when multiple users collaborate sharing a single technical device. The range of technical devices for such a scenario is large, ranging from smartphones on which multiple observers access the same information simultaneously to complex multi-touch tables which are explicitly designed to serve as a collaboration tool for co-located groups.

With regard to the perception and recognition of objects, a substantial body of previous research has confirmed the existence of *canonical viewpoints* from which depicted information can be accessed the most efficiently in terms of errors and speed. Deviations from this canonical viewpoint (i.e., non-canonical views) typically come along with increasing access costs (Diwadkar & McNamara, 1997; Palmer et al., 1981; Tarr, 1995).

**Fig. 1:**
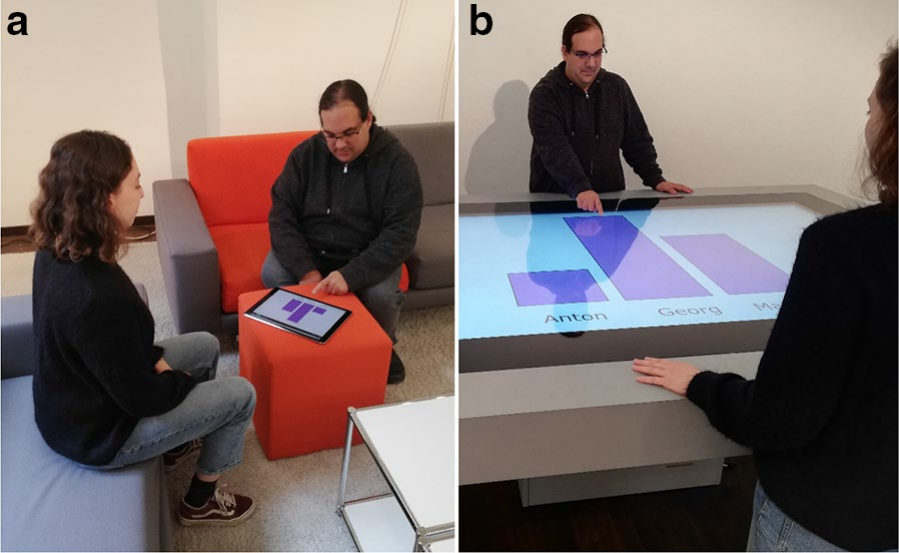
Illustration of the collaborative settings with shared displays. **a** Two users study the same information on an iPad. **b** Two users collaborate using a multi-touch table, an interactive tool for group collaborations. In both scenarios, the display is rotated for one of the observers

With regard to the collaborative view on the bar graphs depicted in Fig. 1, the canonical view is the upright presentation of the graph. Even in rather simple scenarios with only two users such as those depicted in Fig. 1, the viewpoint of one of the observers will deviate from the canonical upright view, as collaborators tend to position themselves on distinct sides of the display (Tang et al., 2006). In other words, (at least) one of the collaborators will have a non-canonical view, which is suboptimal for accessing the displayed information. In the present project, we studied how deviations from non-canonical views decrease the accessibility of the depicted information. Following an approach of use-inspired research, our aim was to investigate ways to reduce the detrimental effects of non-canonical viewpoints based on psychological theories of feature and information processing.

### Information visualization

With the tremendous increase in data and accessibility of data starting in the late twentieth century, data presentation has become an important topic of research, as data visualizations can communicate patterns in data more clearly, efficiently, and concisely than other presentation formats (Cattaneo et al., 2007; Dambacher et al., 2016). In the present project, we chose to study bar graphs as one type of data visualization which is commonly used to summarize frequentist information and therefore reflects a plausible representation of data in collaborative group settings. The challenge of designing intuitive (Neurath, 1936) as well as effective visualizations (Cleveland & McGill, 1984) of data has been investigated across scientific disciplines (e.g., Montello, 2010; Stieff, 2007). The topic of optimizing data visualization has also gained attention from cognitive psychologists (Rensink, 2017; Rensink & Baldridge, 2010), and basic theories on human perception have influenced guidelines for the design of data visualizations (e.g., Evergreen, 2017). A good example of a psychological theory informing data-visualization designers is the feature integration theory (Treisman & Gelade, 1980), which describes a set of basic features that human observers can process without strict capacity limitations. These basic features therefore reflect useful features for graphs (Nothelfer et al., 2017). Another good example is the “picture superiority effect” (Cattaneo et al., 2007; Maisto & Queen, 1992; McBride & Dosher, 2002; Standing, 1973; Whitehouse et al., 2006), which describes the phenomenon that pictures are easier to learn (and retrieve) than words. In the visualization literature, this finding matches the ISOTYPE (**I**nternational **S**ystem **o**f **Ty**pographic **P**icture **E**ducation) guidelines. Emerging from early work of Neurath (1936; see also Neurath & Odgen, 1937), this framework suggests using pictographs rather than written labels to represent units, concepts, and frequencies in visualizations. This framework has received empirical support from a study by Haroz et al. (2015), who showed that pictographs indeed improve information processing relative to simple bar graphs with written labels. Whereas these approaches have made substantial progress in improving the accessibility of visualized information for individual observers, hardly any research has attempted to optimize visualizations for non-upright viewing conditions. This is not too surprising, as the pressing need for such research mostly arose with relatively new presentation technology, such as multi-user touch-tables. However, a substantial body of cognitive psychology research has investigated the question of how human observers access and compare visual information from different viewpoints, such as in the case of image or display rotations.

### Accessing rotated information

Accessing visualized data and viewing and recognizing depictions, representations, and text from unusual angles are common tasks in everyday life. Aside from reading inverted news articles, this encompasses activities such as trying to orient oneself on a city map (Aretz & Wickens, 1992) or reading shop signs on a window from the inside of the shop. In some cases, failure to correctly identify two objects as the same or different from different viewpoints could have serious consequences, for instance, chemists map depictions of molecules (Stieff, 2007) in order to identify enantiomers, which could make the difference between a cure and a poison. Further, it is also a key skill for a physician’s success in laparoscopic surgery (Conrad et al., 2006). Different mental processes have been suggested for accomplishing the mapping operation between rotated views as well as for accessing information from non-canonical viewpoints (see Peissig & Tarr, 2007, for a review). In their seminal work, Shepard and Metzler (1971) asked participants whether two cube structures, which were presented from different viewpoints, were identical or mirrored images of each other. They observed a linear relationship between the time that is necessary for an accurate decision and the angular disparity between both cube structures. From these results, they inferred a mental rotation process (with a constant rotation speed) to align the different views for comparison. The capacity of this mental rotation process, however, remains debated, with some researchers arguing for a holistic rotation of the stimulus (Cooper & Podgorny, 1976), whereas other researchers have reported evidence for piecemeal rotations suggesting a limited capacity (Just & Carpenter, 1985; Yuille & Steiger, 1982; see also Xu & Franconeri, 2015). Whether mental rotation tasks are solved using holistic or piecemeal strategies depends on various factors, such as mental rotation ability (Khooshabeh et al., 2013), sex (Heil & Jansen-Osmann, 2008), and stimulus familiarity (Bethell-Fox & Shepard, 1988). Further, stimulus attributes such as the compressibility of the depicted information influence the amount of simultaneously rotated information (Meyerhoff et al., 2021).

A central limitation in attributing the linear increase in response latencies to a mental rotation process, however, is that such linear relationships also arise in more generalized theoretical conceptualizations of the mapping of information across different viewpoints (Jolicoeur, 1990; Tarr, 1995; Tarr & Pinker, 1989). Among others, these conceptualizations encompass an internalization of 3D-models (Marr, 2010; Marr & Nishihara, 1978), an edge-detection mechanism (Biederman, 1987), or an internalization of multiple viewpoints (Tarr & Bülthoff, 1998; Tarr & Pinker, 1989). In particular, there is neuroscientific evidence that distinct mental operations compensating for deviations in the viewpoint (indexed by distinct neural activity) can hardly be distinguished on a purely behavioral level, as they result in indistinguishable response patterns such as increases in response latency (Gauthier et al., 2002). The goal of our present research is not to disentangle the different mental processes contributing to the compensation of deviations in viewpoint. Derived from the work of Risko et al. (2014), we will therefore use the theoretically neutral term *mental normalization* as an umbrella for all mental operations contributing to solving this task. While the ubiquitous occurrence of linear decreases in performance makes it difficult to isolate particular mechanisms of mental normalization, it also emphasizes that—irrespective of the exact underlying mechanism—the costs that come along with deviating viewpoints likely affect a substantial number of practical tasks.

In the present experiments, we studied mental normalization for information summarized in bar graphs. Although we are not aware of any direct test of viewpoint costs on accessing information in bar graphs, there are good reasons to propose that these costs follow the same pattern as all previously described tasks. Most relevant here are observations demonstrating that individual elements of bar graphs themselves are subject to costs of mental normalization when their presentation deviates from upright viewing conditions. For instance, such detrimental effects have been consistently reported for shape information (Cooper, 1975), cube figures (Shepard & Metzler, 1971), letters (Corballis & McLaren, 1984; Rüsseler et al., 2005), words (Koriat & Norman, 1985), sentences (Risko et al., 2014), and pictures (Tarr & Pinker, 1989). As most of these effects were observed in prolonged response latencies, it appears likely that extracting information from bar graphs is also prolonged when the presentation deviates from the upright view.

In our experiments, we followed a use-inspired rationale. First, we aimed to establish the costs of mental normalization for accessing information depicted in bar graphs. Second, we strove to understand whether such detrimental effects arise from the rotation of the graph itself or merely due to the rotation of written labels. Third, and finally, we aimed at reducing the costs of mental normalization for information depicted in bar graphs by transferring effects from knowledge-driven basic psychological research into our use-inspired scenario. We arranged our experiment to closely resemble the situation of a collaborative setting as depicted in Fig. 1. For instance, the participants solved the task on a horizontal touch display which has the same functionality as a smartphone or a multi-touch table. Nevertheless, we tested participants individually in order to allow us to isolate the effect of display rotations with full experimental control.

## Experiment 1

**Fig. 2:**
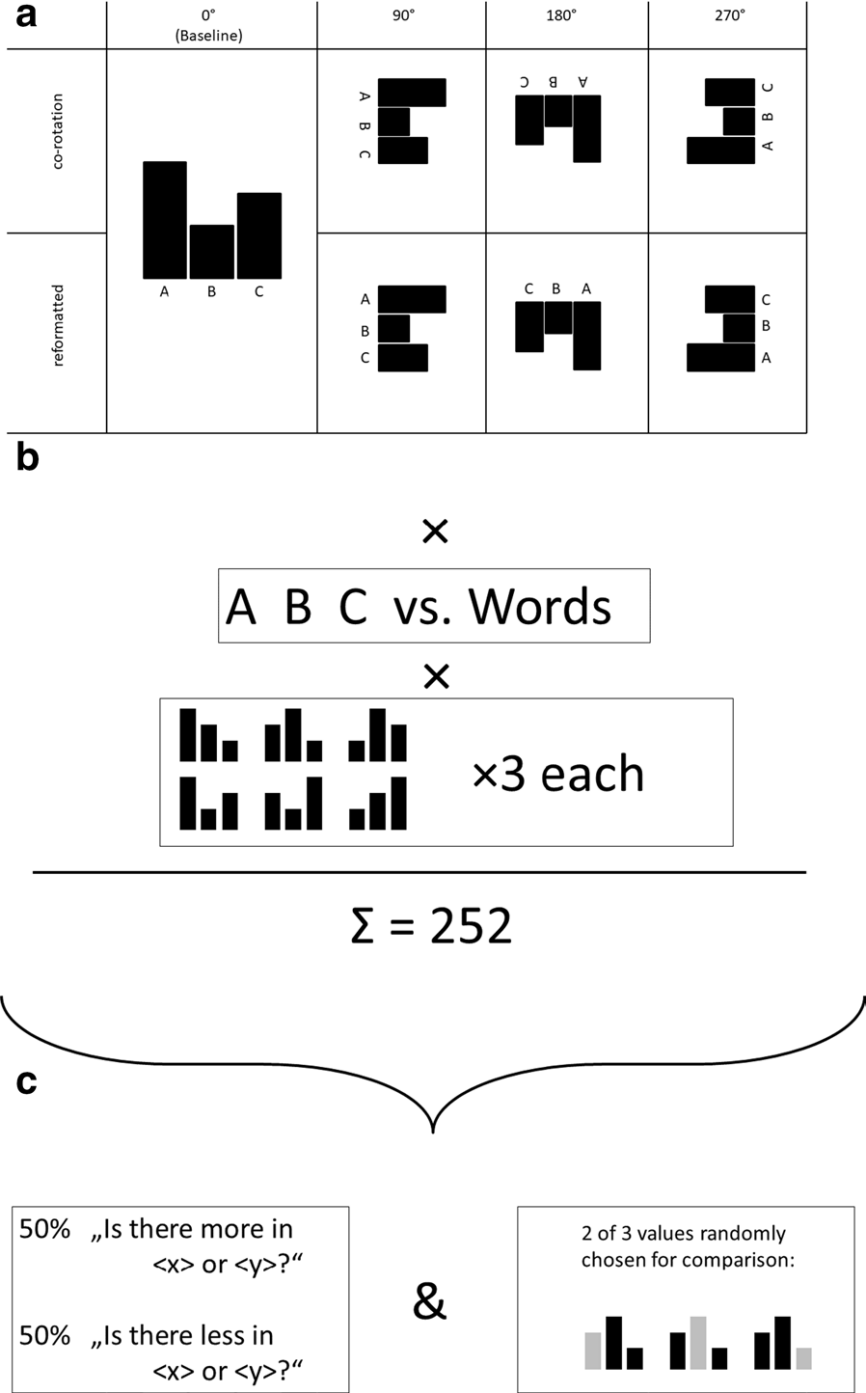
Illustration of the design of Experiment 1. **a** The baseline condition is an unrotated bar graph labeled either with words or letters. Relative to this baseline, we investigated three different angular rotations (90°, 180°, and 270°) and two different categories of labels (co-rotating vs. reformatted). **b** We manipulated the labels of the bar graph (words vs. letters). There were six different arrangements of the bars (each repeated three times), resulting in a total of 252 trials. **c** In one half of the trials (selected randomly), the participants had to indicate the smaller bar in comparison. In the other half, the participants indicated the larger bar. The two bars involved in the comparison were selected randomly. Each participant received a unique set of stimuli

With the first experiment, we aimed to demonstrate that rotated views of a bar graph interfere with the extraction of the depicted information (i.e., the costs of mental normalization). We focused mainly on response latency as a proxy for task performance with prolonged response latencies signaling the proposed interference. We asked our participants to answer comparative questions by extracting the corresponding information from the bar graph. We hypothesized that a rotated presentation of the bar graph would elicit longer response latencies. A rather obvious candidate for the expected detrimental effect of graph rotation on response latencies is the written labels which identify the individual bars. To investigate the impact of such labels further, we introduced two additional manipulations. First, we compared words versus letters as labels. We expected that the more simplistic shapes of the letters could be identified faster across rotations, thus reducing the detrimental effects of display rotations. Second, we compared two variants of presenting the labels: co-rotation in which the labels rotate with the bar graph (similar to a physical rotation of a sheet of paper) versus reformatted labels which maintain their upright orientation relative to the observer (see Fig. 2a for an illustration). If the detrimental effect of display rotations on response latencies emerges only from difficulties in recognizing the rotated labels, the conditions with reformatted labels should not differ from the unrotated view.

### Methods

#### Power considerations

The most relevant manipulations in our experiments were the label rotation scheme (reformatted vs. co-rotated labels) and the label type (e.g., different labels such as text and letters in Exp. 1). Introspectively, both manipulations had an influence on response onsets, suggesting substantial effect sizes (*η*_*p*_^2^ > 0.25). However, there was no directly related prior work that could have served as a more precise estimate for effect sizes. The closest related study investigated response latencies for rotated textual stimuli (Risko et al., 2014) and observed large effect sizes (*η*_*p*_^2^ = 0.81 in their Experiment 1; *η*_*p*_^2^ = 0.68 in their Experiment 2). However, this study did not involve representations of data such as ours so that these estimates are presumably too large. Consequently, as our study is the first of its kind, we intended to power it appropriately for lower effect sizes (*η*_*p*_^2^ = 0.10; assuming correlations among repeated measures of *r* = 0.50). A corresponding power analysis (1 − *β*) > 0.95 at *α* = 0.05) suggested a minimum sample size of 32 participants (G*Power, Faul et al., 2007). In order to compensate for potential data exclusions, we slightly overpowered this sample size, resulting in 33 participants in Experiment 1 and 35 participants in Experiment 2. In Experiment 3, we unintentionally overrecruited the sample, resulting in 41 participants.

#### Participants

Thirty-three students (22 female, 18–35 years) from the University of Tübingen, recruited via an online platform for volunteer participants for experiments, took part in Experiment 1. They received a compensation of 5€ for 40 min of their time. The experimental procedure was ethically approved by the institutional review board of the Leibniz-Institut für Wissensmedien, Tübingen, and all participants provided informed consent prior to testing.

#### Apparatus

The experiment was conducted on a horizontal 23″ touch sensitive monitor (Dell Panel Monitor S2340Tt) controlled by a HP Elitebook 8530p. The experimental scripts were coded in Python using the PsychoPy libraries (Version 1.85.1; Peirce, 2007, 2008). The unrestricted viewing distance was approximately 60 cm.

#### Materials and procedure

**Fig. 3:**
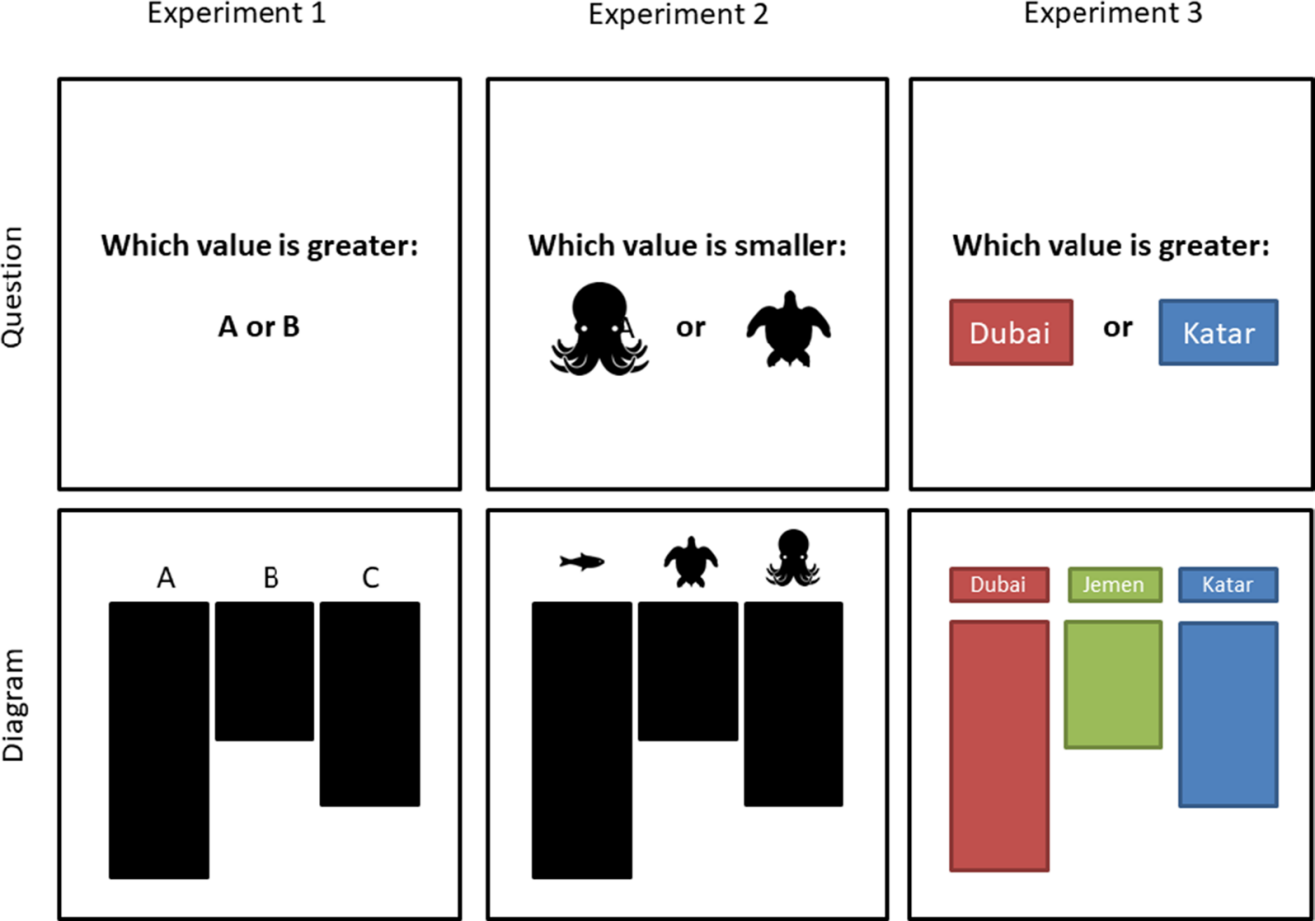
Schematic depiction of the stimuli for the reported experiments. Experiment 1 used letter and word labels. Experiment 2 employed word and pictograph labels. In Experiment 3, the coloring of the bars was manipulated. All bars had either the same or different colors

Our participants solved a series of 252 simple questions for which the answers were depicted in a bar graph (see Fig. 3). Each trial started with a question informing the participant of the names of two values that she/he should subsequently compare (e.g., “Is there more in A or B?” or “Is there less in A or B?”). This question was presented in font size 40 in the middle of the screen. The participants preceded with the trial by putting their index finger down onto the start position, which was horizontally in the middle of the screen, approx. 6.7 cm from the bottom, and 4.5 cm in diameter. Next, a bar graph (15.2 × 15.2 cm), consisting of three bars, appeared in the center of the screen. Two of the bars corresponded to the values that the participants were asked to compare. The third bar corresponded to the values of a related category but was irrelevant for answering the question. It was added so that the participants would have to identify and select the relevant bars first (see Fig. 2c).

All bars clearly differed in height, as all bars had a constant height and constant height differences, with a bar height pool of 3.1 cm, 7.1 cm, and 10.9 cm. To rule out potential influences from the relative bar positions, we presented all possible permutations of the bars in each condition to each participant. The bars that we asked participants to compare were chosen randomly. In one half of the trials, the participants were asked to choose the larger of the two values. In the remaining half of the trials, the participants had to choose the smaller of the two values (see Fig. 2c). We randomly generated a new set of trials for each participant.

We manipulated the presentation of the bar graph. First, we manipulated the orientation of the bar graph itself (0°, 90°, 180°, and 270°, clockwise with 0° being the standard upright bar graph view). Second, we manipulated whether the labels of the bars rotated with the bars (co-rotate) or whether they remained upright to the participant (reformatted). Third, we manipulated whether the labels of the bars consisted of words or letters. We sampled the word labels from a list of 20 topics containing three labels each (e.g., topic “male first names” with the labels “Anton,” “Malte,” and “Georg”). To control for word length, we included only words with two syllables consisting of a total of five letters. In the condition with letters as labels, the letters were randomly sampled from the following 17 letters: A, B, C, F, G, H, I, K L, O, R, S, T, U, W, X, Z. The remaining letters were excluded as their appearance is too similar to other letters when being rotated.

**Fig. 4:**
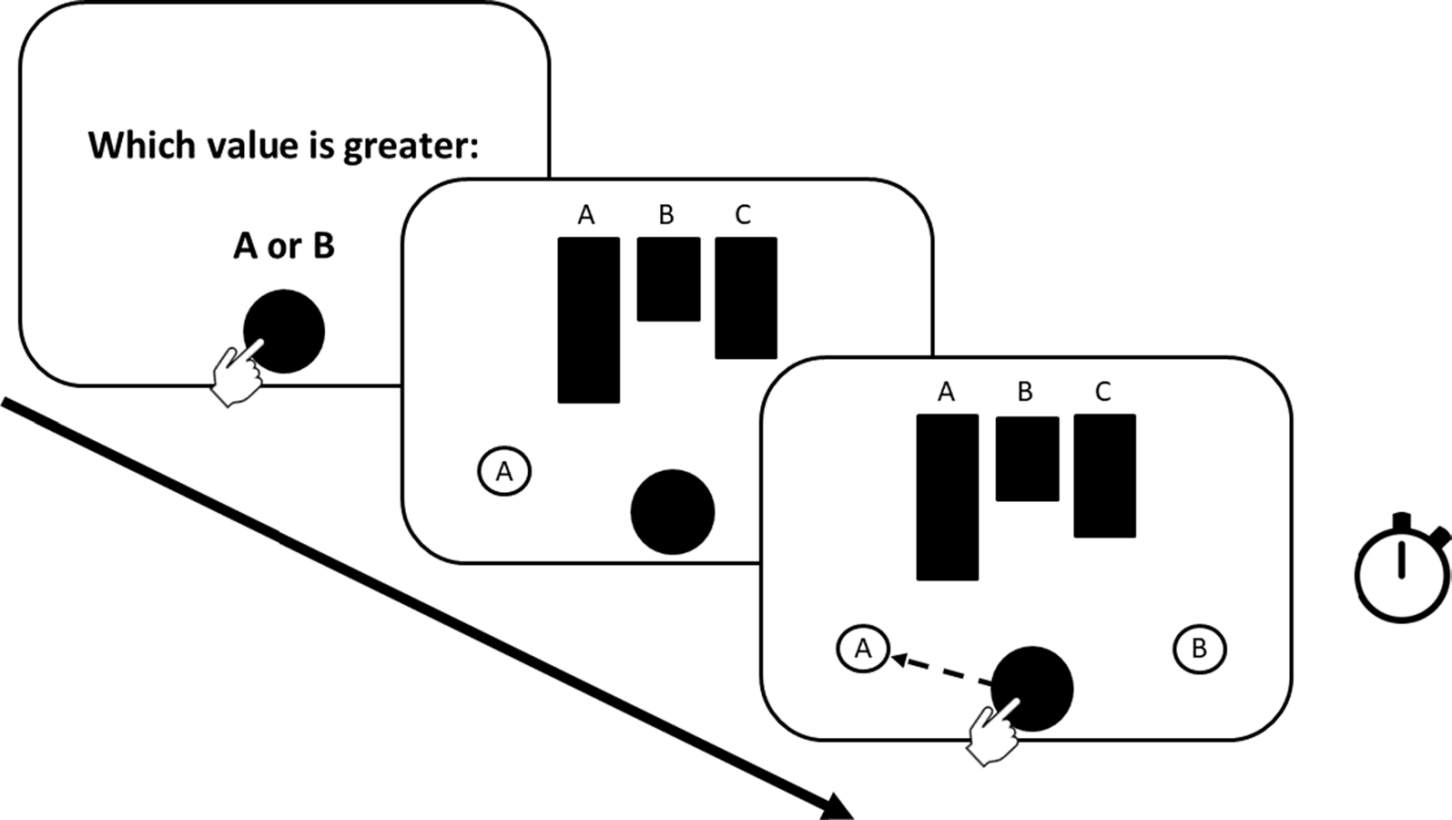
Experiment 1 flow diagram. For each trial, participants were first presented with a question. After putting their finger on the button, a diagram for answering the question appeared. Participants gave their response by sliding the button onto one of two options. The time to start the movement of the correct trials was taken as the dependent variable

The participants responded by moving their finger to one of two response boxes below the bar graph, each of which corresponded to one of the values in question. The response boxes were positioned at 9.5 cm from the bottom and 20.2 cm from the left and right side of the screen and measured 2.4 cm in diameter. As the dependent variable, we captured the latencies of the initiation of a response by the participants (i.e., the time difference between onset of the bar graph and the start of the movement of the index finger towards the response box). For an illustration of the trial setup, see Fig. 4.

#### Design

All factors were manipulated in a within-subject design. Please note that in the 0° rotation condition both the reformatted and the co-rotated text looked identical. Therefore, this condition serves as a baseline. The response latencies of this baseline are subtracted from each other condition in order to isolate the effect of the rotation. The remaining combinations follow a 3 (angular rotation: 90°, 180°, 270°) × 2 (label rotation scheme: reformatted vs. co-rotated text) × 2 (label type: words vs. letters) within-subject design. Each factor combination was repeated 18 times (including three repetitions of each permutation of the bar heights), cumulating to 252 trials per participant (see Fig. 2a, b).

#### Analysis plan

Our research questions focused on the relative difference between conditions with rotations of the bar graphs and the baseline condition without such rotations. In order to isolate these effects of the rotation, we subtracted the mean of the 0° rotation condition (individually for each participant). Our analyses of the data focused on two questions. First, we analyzed how label rotation scheme and label type affected performance across the remaining angular rotations. For this analysis, we conducted a repeated measures ANOVA on the differences in response latencies between the conditions with rotation and the baseline without rotation. Second, we analyzed whether the conditions with rotations differ from the baseline without rotation (i.e., whether the difference scores differ from zero). We tested this with a series of *t*-tests. As analyses with multiple tests are prone to alpha error cumulation, we used a Bonferroni-corrected alpha level of *p* = 0.00416 for this series of 12 *t*-tests. Please note that this is a rather conservative correction which comes along with the risk of incorrectly classifying meaningful results as insignificant. The challenge for the current project was that insignificant deviations from the baseline might signal the successful prevention of prolonged response latencies; we therefore attempted to prevent that such a conclusion would only be based on insignificant tests. Beyond pure significance, we therefore also considered effect sizes as an indicator for the relevance of a particular result.

In accordance with Cohen (1969, p. 25), we considered effects with a size of *d*_*z*_ < 0.2 as negligible.^1^ Therefore, for evaluating the reduction in costs associated with rotations, we only interpreted insignificant results with *d*_*z*_ < 0.2 as a successful reduction in rotation costs, whereas the results which were insignificant (after the correction) with *d*_*z*_ > 0.2 cannot be interpreted either way.

### Results

Overall, accuracy was very high (*M* = 95.42%, SD = 3.1%). Accuracy levels for all experimental conditions ranged from 94.8% to 95.9%. These discrepancies were deemed negligible. For the analysis of response latencies, all trials with incorrect responses were removed from the data set. Further, we excluded trials for which we registered the motion onset of the index finger less than 250 ms after stimulus onset, as they likely reflect anticipations, involuntary movements, and measurement errors rather than regular task processing (6.46%) as well as trials of which the response latency deviated more than 3 SD from the personal mean (i.e., outliers, 1.32%).

Following the exclusions, we calculated the difference score in response latencies between the experimental conditions with rotations and the baseline without rotation (raw response latency data for all three experiments are available in Table 1 in the Appendix). Finally, we aggregated this difference score for all conditions separately for each participant.

**Fig. 5:**
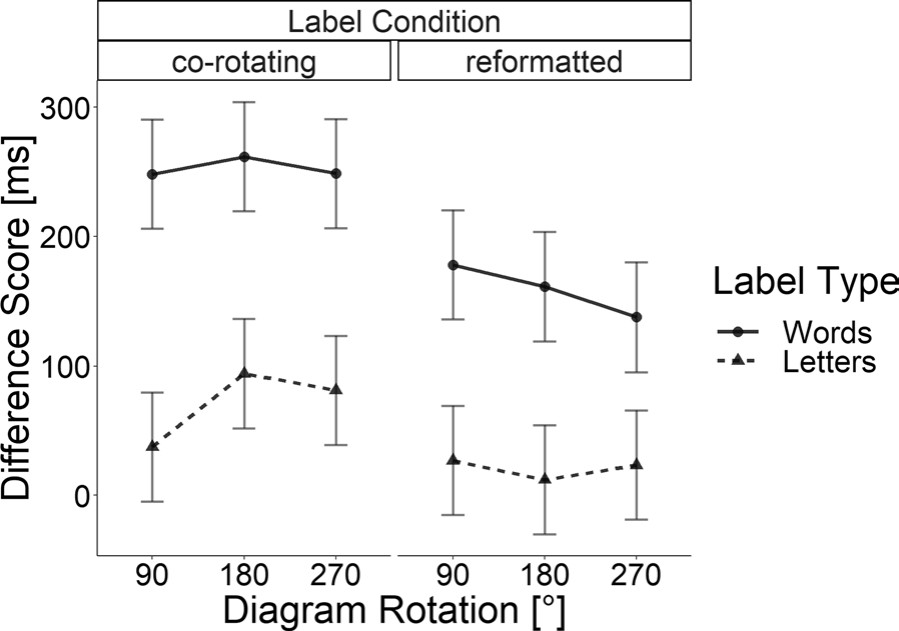
Results of Experiment 1. Mean differences of the response onset relative to the baseline without rotation. Error bars are based on Fisher’s Least Significant Difference (LSD; Fisher, 1935)

In order to examine differences between the conditions with rotations, we conducted a repeated measures ANOVA with angular rotation (90°, 180°, 270°), label rotation scheme (co-rotating, reformatted), and label type (word, letter) as the independent variables, as well as the difference score in response latency as the dependent variable. This analysis revealed a main effect of the label rotation scheme, *F*(1, 32) = 16.19, *p* < 0.001, *η*_*p*_^2^ = 0.34, indicating larger deviations of the response latencies from those of the baseline when the label orientation rotated with the bar graph rather than when it remained upright relative to the observer (see Fig. 5). Further, we observed a main effect of the label type, *F*(1, 32) = 49.21, *p* < 0.001, *η*_*p*_^2^ = 0.61, indicating that words revealed larger deviations from the baseline than letters. There was no main effect of the three different angular rotations of the bar graph, *F*(2, 64) = 0.11, *p* = 0.89 and neither the two-way interactions, all *F*s(2, 64) < 1, all *p*s > 0.376 and *F*(1,32) = 1.723, *p* = 0.198, nor the three-way interaction, *F*(2, 64) = 0.137, *p* = 0.872, reached significance.

Furthermore, we compared the difference scores of all rotation conditions to zero with a series of *t*-tests (Bonferroni-corrected *p* = 0.00416). All conditions in which words served as labels showed significant differences to the baseline (all *t*s(32) > 3.64, all *p*s < 0.001, all *d*_*z*_s > 0.634). For the conditions in which letters served as labels, none of the *t*-tests showed significant difference (after the corrections) to the baseline (all *ts*(32) < 2.32, all *ps* > 0.02). The effect sizes for letter label conditions varied: Reformatted letter labels showed negligible effects (all *d*_*z*_*s* < 0.122), while co-rotated letter labels showed small effect sizes (all *d*_*z*_*s* > 0.21 and < 0.41).

### Discussion

The results of this experiment highlight that the written label provided a central challenge in accessing information from rotated bar graphs. Labels consisting of written words (rather than letters) that co-rotated with the bar graph (rather than being reformatted) revealed the largest mental normalization costs. The attribution of normalization costs to written labels is further supported by the results in the conditions with letters as labels. In particular, we did not observe substantial costs when the letters remained upright to the observers, signaling that it was not the rotation of the bars that induced the mental normalization costs. These results are particularly challenging, as written labels that co-rotate with the bar graph reflect the most authentic instance of mental normalization “in the wild,” as reformatted labels cannot be realized for more than one viewer, and letters are hardly ever appropriate descriptions of depicted data. In the following experiments, we therefore implemented modifications of the labels and the presentation of the bar graph that were intended to improve the accessibility of the depicted information during rotated views.

## Experiment 2

As Experiment 1 revealed that word labels seem to be the Achilles’ heel of mental normalization processes for information depicted in bar graphs, we explored the potential of pictorial labels for overcoming such normalization costs. We opted to try pictographs, as pictures seem to have a general superiority for information transportation. They are more accurately remembered than words (Cattaneo et al., 2007; Standing, 1973). Furthermore, response latencies are shorter for pictures than for words (Jenkins et al., 1967; Shor, 1971). In the context of information visualization, ISOTYPE-esque pictographs have been demonstrated to promote the understanding of bar graphs (Haroz et al., 2015). These studies suggest that pictures may act as good substitutes for word labels in data visualizations. In Experiment 2, we therefore explored whether the benefit of pictographs as labels for bar graphs also applies to mental normalizations. Although the costs of mental normalization for image stimuli have been demonstrated previously (Quaiser-Pohl, 2003), it remains possible that these costs are less pronounced for very simplistic pictographs (for an example of the pictograph label condition, see Fig. 3). Also, it is unclear how potential mental normalization costs of pictograph labels relate to those of written labels. As in Experiment 1, we manipulated whether the label rotated with the bar graph or whether it maintained its orientation relative to the observer. If normalization costs arise from the rotation of the labels, the conditions with reformatted labels should be at the level of the unrotated baseline (i.e., a difference score around 0 ms). Finally, we again manipulated the angular disparity between the presented bar graph and its upright orientation. Although this manipulation had no effect in Experiment 1, we decided to maintain this manipulation for exploratory purposes as well as consistency across experiments.

## Methods

### Participants

Thirty-five new students (28 female) from the University of Tübingen (18–33 years), recruited via an online platform for volunteer participants in studies, took part in this experiment. They received 5€ for approximately 40 min of their time.

### Stimuli and procedure

**Fig. 6:**
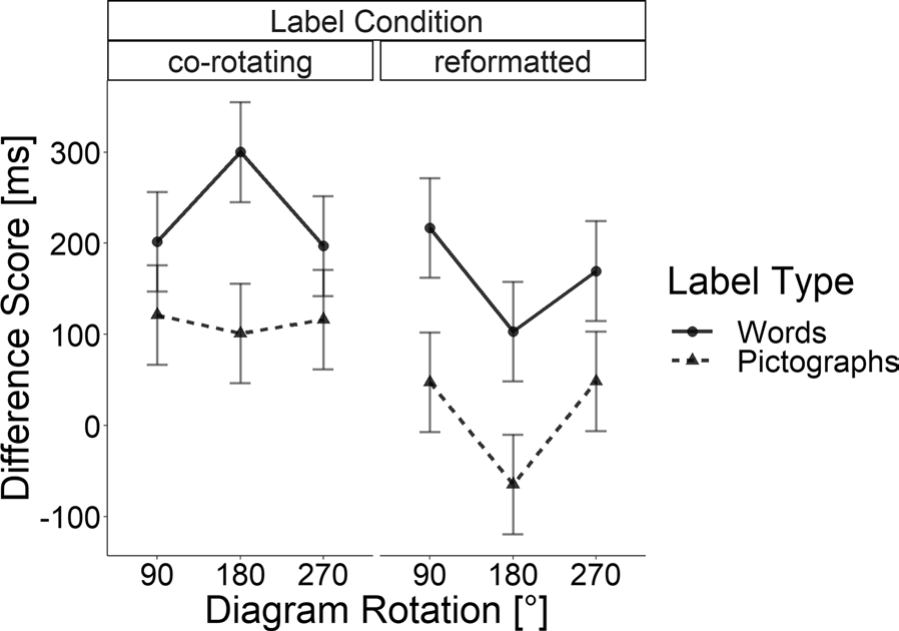
Results of Experiment 2: Mean differences in response onset relative to the baseline without rotation. Error bars indicate Fisher’s Least Significant Difference

All stimuli and procedure were identical to the first experiment with the following exceptions: As label types, we compared written words with pictographs as labels (see Fig. 6). We used the pictographs reported in the study by Haroz et al. (2015). The written labels were chosen to match the meanings in the pictographs.

### Design

The experiment followed a 3 (angular rotation: 90°, 180°, 270°) × 2 (label rotation scheme: reformatted vs. co-rotated text) × 2 (label type: words vs. pictographs) within-subject design. As in the first experiment, we subtracted the baseline without any rotation from all other conditions in order to isolate the effect of display rotation.

### Results

The overall subject accuracy was 94.37% (SD = 4.6%). Accuracy levels for the different factors fell between 93.65 and 95.16%. Trials with a motion onset of the index finger below 250 ms were excluded (8.7%) as were outliers (1.4%). The analysis plan was identical to Experiment 1.

In order to examine differences between the conditions with rotations, we conducted a repeated measures ANOVA with angular rotation (90°, 180°, 270°), label rotation scheme (co-rotating, reformatted), and label type (words, pictographs) as the independent variables, as well as difference scores in response latency as the dependent variable. Again, the rotation of the bar graph did not create a significant increase in the difference score, *F*(2, 68) = 0.94, *p* = 0.39. However, the label type showed a significant difference, *F*(1, 34) = 22.42, *p* < 0.001, *η*_*p*_^*2*^ = 0.40, indicating larger difference scores in response latencies for text than for pictographs. Further, there was an effect of the label rotation, *F*(1, 34) = 12.71, *p* = 0.001, *η*_*p*_^*2*^ = 0.272, indicating larger difference scores with co-rotated than with reformatted labels. No interaction reached significance with *F*s(2, 68) < 2.48, *p*s > 0.091 and *F*(1, 34) = 1.03, *p* = 0.317. The results are depicted in Fig. 6.

Furthermore, we tested whether the difference score in response latencies deviated from zero with a series of Bonferroni-corrected *t*-tests (*p* = 0.00416). In the word condition, the difference score for the word labels that maintained their upright orientation to the observer in the 90° and 270° diagram rotation condition did not reach significance with *t*(34) = 1.825, *p* = 0.076, *d*_*z*_ = 0.31 and *t*(34) = 2.73, *p* = 0.01, *d*_*z*_ = 0.46, respectively, whereas the remaining four conditions reached significance, all *ts*(34) > 3.12, *ps* < 0.0037, all *d*_*z*_*s* > 0.527. In the conditions with pictorial labels, none of the tests reached significance, all *ts*(34) < 2.03, all *ps* > 0.05. The stably displayed pictograph labels showed negligible effect sizes with all *d*_*z*_*s* < 0.2, while the co-rotated pictograph labels showed small effect sizes (0.28 < all *d*_*z*_*s* < 0.34).

### Discussion

While the rotation of the diagram itself did not cause significant increases in response time relative to the unrotated condition, rotating the label did. Consistent with Experiment 1, there was a significant difference between reformatted and co-rotated labels, signaling that identifying the labels from rotated views was the weak spot in accessing depicted information from rotated bar graphs. With regard to the label type, there was a significant difference between word labels and the corresponding pictograph labels. Pictograph labels deviated less from the unrotated baseline. Furthermore, the comparison of each condition with the baseline (i.e., the difference score) showed a similar pattern of Cohen’s d values as in Experiment 1. In particular for the pictographic labels, only the conditions with stably oriented labels to the observer revealed negligible effect sizes, whereas there remained substantial effects sizes for the conditions in which the pictographs rotated with the bars of the graph. As reformatted labels cannot be realized for more than one viewer at a time, using pictographs as labels is therefore not sufficient to (fully) compensate for the effects of mental normalization in bar graphs.

## Experiment 3

In this experiment, we tested the potential of colored text labels for reducing the detrimental effects of graph and label rotation. As color is considered to be processed efficiently (i.e., in parallel for the entire display) (Treisman, 1986), coloring labels might not impose an additional load on working memory but may support a faster mental normalization instead. An example for the color intervention stimuli is given in Fig. 3, Experiment 3.

### Methods

#### Participants

Forty-one students (33 female) from the University of Tübingen, aged 18–27 and recruited via an online platform for voluntary student participants, took part in this experiment and were paid an allowance of 8€ for 40 min of their time.

#### Stimuli and procedure

Stimuli and procedure were identical to Experiment 1 with the following exceptions: The label type factor consisted of a “multicolor” and a “monocolor” condition. In the multicolor condition, each bar was filled with a different color and the associated word labels were highlighted in the same color, in the question as well as in the diagram (see Fig. 6). The colors were selected randomly in order to avoid systematic influences from semantic associations between the selected color and the corresponding label (Lin et al., 2013). In the monocolor condition, all bars were filled with the same color and their labels were not highlighted.

With regard to the monocolor condition, we expected to replicate Experiment 1: Diagrams with co-rotating labels should show longer response latencies than diagrams with reformatted labels (and all conditions should differ from the baseline). Further, we expected the multicolor condition to (at least partially) reduce the costs of mental normalization. Furthermore, we explored how this expected reduction in mental normalization costs relates to the baseline without rotations.

### Results

The overall subject accuracy was 94.9% (SD = 3.1%). Accuracy levels of the factors were between 93.43 and 95.49%. Trials with a motion onset of the index finger below 250 ms were excluded (9.5%) as were outliers (1.5%).

**Fig. 7:**
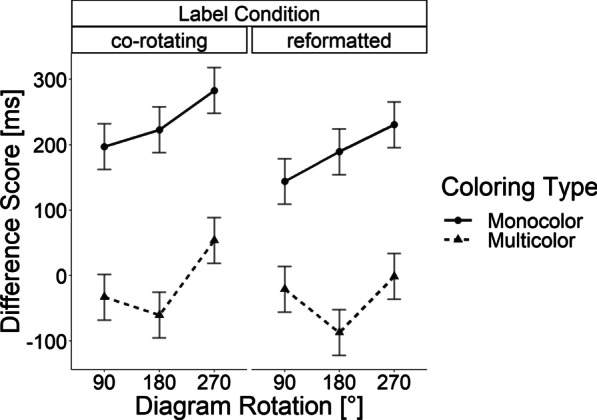
Results of Experiment 3: Mean differences of the response onset relative to the baseline without rotation. Error bars are based on Fisher’s Least Significant Difference

Analogous to the previous experiments, we conducted a repeated measures ANOVA with three factors: angular rotation (90°, 180°, 270°), label rotation scheme (co-rotating, reformatted), and coloring type (monocolor, multicolor). The difference score in response latency was again used as the dependent variable. In contrast to Experiments 1 and 2, the label-rotation did not reach significance in this experiment, *F*(1, 40) = 2.91, *p* = 0.096. However, the coloring type yielded significant differences, *F*(1, 40) = 57.05, *p* < 0.001,, *η*_*p*_^*2*^ = 0.588, indicating a reduced deviation from the baseline for multicolor than monocolor labels. Finally, the difference score in response latencies increased with angular deviation in this experiment, *F*(2, 80) = 10.11, *p* < 0.001, *η*_*p*_^*2*^ = 0.202. None of the two- or three- way interactions reached significance, *Fs*(2, 80) < 2.69, all *ps* > 0.07 and *F*(1, 40) = 1.09, *p* = 0.302. The results are depicted in Fig. 7.

To further explore these results, we tested the difference score in response latencies against zero with a series of Bonferroni-corrected *t*-tests (*p* = 0.00416). The difference scores for response latencies reached significance in all monocolor conditions, all *t*s(40) > 4.925, all *p*s < 0.0001, all *d*_*z*_*s* > 0.76. However, the difference score did not reached significance in any of the multicolor conditions, all *ts*(40) < 1.5, all *ps* > 0.029. All effect sizes but one were negligible with all *d*_*z*_*s* < 0.2. Only the multicolor condition with 270° diagram rotations and co-rotated word labels showed a small effect size with *d*_*z*_ = 0.237.

### Discussion

Contrary to the previous experiments, the rotation of the label had no significant effect on response latencies in this experiment. Numerically, it seems as if there was a small effect on the label rotation scheme in the monocolor condition but clearly absent in the multicolor condition. However, as there was no significant label type x label rotation scheme interaction, such a conclusion cannot be drawn directly from the data of this experiment. What also deviates from the preceding experiments is that there was an effect of the angular rotation of the graph, which we did not observe in the previous experiments. A (rather speculative) explanation for this is that the coloring reduced the contrast between the labels and the background, possibly having reduced the readability of the labels. In return, this might have induced additional normalization costs which might increase with the angle of rotation. Nevertheless, what is consistent with the preceding experiments (numerically as well as statistically) in this experiment is that in the multicolor conditions, the difference score in response latencies hardly increased with rotations of both graphs and labels (in terms of negligible effect sizes). Multicolored bars and labels therefore appear to be a promising approach in reducing the costs of mental normalizations in bar graphs.

## General discussion

In this study, we investigated how non-upright viewing conditions impact the extraction and processing of information from bar graphs. We followed a use-inspired research approach by converting a practical problem (i.e., that only one user could have upright viewing conditions on shared graphs in collaborative settings) into a laboratory research paradigm.

A first major result of our study is that bar graphs in non-upright viewing conditions indeed pose an additional burden on cognitively processing the depicted information. In all three experiments, the standard condition (i.e., word labels) yielded significant normalization costs (i.e., the difference in response latency relative to the baseline). With regard to our practical collaboration setting outlined in the introduction (see Fig. 1), this finding confirms that in most cases a collaborator with non-upright viewing conditions would have to invest additional cognitive resources in order to access the same information as the other collaborator with upright viewing conditions.

A second major result of our study is identifying the rotation of the labels as the “Achilles’ heel” in terms of normalization costs for compensating for non-upright viewing conditions. This conclusion stems from the observation that normalization costs were remarkably reduced (in most cases to negligible effect sizes) when the labels maintained their upright orientation relative to the observer. Whereas these conditions with reformatted labels are helpful in identifying the origin of the largest proportion of the normalization costs, it appears unlikely that this finding could be part of an intervention aiming to reduce the normalization costs. The reason for this is because maintaining the upright orientation of the labels can be realized for multiple collaborators only by introducing additional, redundant labels with different orientations so that each observer has his/her own label with an upright orientation. Such an approach, however, would be challenging technically as well as psychologically. From the technical perspective, it would be necessary to track the number as well as the location of collaborators with non-upright viewing conditions and add the necessary labels at their corresponding orientation. While this technical challenge can be met (e.g., Dietz & Leigh, 2001; Ramakers et al., 2012 for multi-touch tables), the psychological challenge is more difficult. Psychologically, the inflation of labels at different orientations would provide an additional burden on the perceptual and cognitive processing of the depicted information. Given the known capacity limitations for attentional processing (e.g., Meyerhoff et al., 2017), it seems unlikely that human observers can deal with the inflation of labels. Consequently, all collaborators would have to ignore the additional labels while focusing only on those labels presented upright from their current point of view in order to reduce the information load. However, an extensive body of research has shown that irrelevant stimuli in the visual field cannot always be ignored even when processing them is harmful for performance in the task at hand (Eriksen & Eriksen, 1974; Eriksen & Schultz, 1979; Kopp et al., 1994):

As an alternative approach to reducing the costs of mental normalization in bar graphs, we therefore investigated two candidate modifications of the labels and/or bars. In Experiment 2, we investigated pictographs as labels for which we observed a substantial reduction in normalization costs. However, in particular when the pictographs rotated with the bar graph, the effect sizes were—at least for the critical “co-rotation” condition—not in the negligible range (although not significant due to the Bonferroni-corrections). Please note that we changed only the label to its corresponding pictograph, not the bars themselves, as has also been done in previous work (Haroz et al., 2015). It therefore remains possible that changing the bars themselves into pictographs would further diminish normalization costs to the negligible range. However, it appears more likely that pictographs would persist in showing small normalization costs even under these circumstances, as pictures clearly have canonical viewpoints facilitating their identification (Palmer et al., 1981). As picturizing labels are restricted to contents that can be turned into intuitive pictographs, we instead investigated the coloring of bars and labels in the remaining third experiment. In line with the intensive research demonstrating the efficiency of color processing in terms of visual search (Wolfe & Horowitz, 2004, 2017), perceptual grouping (Palmer et al., 2003), as well as attentional efficiency (Carter, 1982; Christ, 1975; Duncan & Humphreys, 1989), we also observed that this coloring manipulation reduced mental normalization costs to the negligible range of effect sizes. In sum, both pictographs and coloring reduce normalization costs, but coloring appears to be slightly more efficient.

Although our experiments were not designed to disentangle different processes involved in the processing of rotated displays, there are two findings in our results which we would like to elaborate. First, in contrast to the many previous studies exploring normalization costs for rotated stimuli (for an overview see Khooshabeh et al., 2013), the angular deviation from upright viewing conditions played only a minor role in our results. Although the rotated display was generally harmful in all three experiments, normalization costs only increased with angular deviation in Experiment 3. The most plausible reason for this reduced relevance of angular rotation is that we studied orthogonal or opposite viewpoints which are known to be less prone to normalization costs than angular deviations between these orientations (Hintzman et al., 1981; Montello, 1991). In the present project, however, we focused on the orthogonal and opposite viewpoints because these seem to be the most common perspectives in the collaborative group settings with multi-touch tables (e.g., Bause et al., 2018; Higgins et al., 2011, 2012; Rogers et al., 2009), which inspired our project.

Second, our results revealed less pronounced normalization costs when the labels remained in their upright orientation to the observer. This observation is consistent with a piecemeal mental normalization strategy rather than a holistic mental normalization strategy. The reason for this is because a holistic mental normalization would result in the opposite effect. Such a holistic normalization would turn the labels away from an upright orientation, thus increasing response latencies. Overall, this finding is consistent with the literature on classical mental rotation which has observed holistic strategies mostly for shape information (Cooper, 1975; Cooper & Podgorny, 1976; Cooper & Shepard, 1973) but not for stimuli that required the integration of multiple visual feature (Hochberg & Gellman, 1977; Xu & Franconeri, 2015).

Given the complexity of a graph (relative to simple shapes), it appears likely that multiple processes contribute to the overall performance as well as the normalization costs. One candidate mechanism that might speed up the processing of the depicted information is feature-based visual search (e.g., Treisman, 1986; Wolfe & Horowitz, 2017). In particular, our final experiment showed that color-coding reduced normalization costs to a negligible range. Combining a selection of relevant information based on color with a piecemeal rotation strategy might allow participants to mentally normalize only relevant information, thus reducing the overall costs. Future research is necessary to disentangle such combined processes of mental normalization. Such a line of research would probably have to combine behavioral and psychophysiological methods in order to isolate the individual components of rather complex mental normalizations (Gauthier et al., 2002). Another promising venue would be to investigate individual differences, as many mental normalization tasks are known to be subject to individual differences arising from spatial abilities (Collins & Kimura, 1997; Khooshabeh et al., 2013; Peters et al., 2006; Tapley & Bryden, 1977; Tarampi et al., 2016), general intelligence (Varriale et al., 2018), or expertise with the stimulus materials (Stieff, 2007). In the present project, we neglected these differences (we drew the sample from our rather homogenous group of students), as we focused on the basic effects that most likely arise across all participants (although they might be differently pronounced). For the same reason, we neglected the differences for sex (Burnett, 1986; Jones & Anuza, 1982; Semrud-Clikeman et al., 2012) and age (Hertzog & Rypma, 1991), which have been reported for many tasks.

## Limitations and outlook

To the best of our knowledge, our project is the first to address the impact of mental normalization as it would appear in collaborative settings dealing with the extraction of depicted information from graphs. Given the novelty of this research approach, there are, of course, limitations which should be further explored in future research. A first limitation addresses the coloring intervention. Although coloring clearly reduced normalization costs, it seems likely that color coding works only for a limited number of depicted elements. With an increasing number of elements in a graph, the colors will, by nature, become more similar in the color space, making it more difficult to distinguish the colors efficiently (Cahill & Carter, 1976). Thus, there will certainly be a threshold after which color coding of graph elements will not be helpful anymore. Furthermore, coloring the bars uniquely becomes more challenging when the bar graph depicts more than one data dimension. As such, bar graphs typically use color or shading information to group the bars. Adding (further) color cues to link the labels with the bars might be confusing. Thus, future research should attempt to replicate the usefulness of the color intervention with more complex data structures. Along these lines, it would be useful to extend the general research approach to other types of data graphs in order to ensure that the normalization costs that we observed in the present experiments, as well as the success of the interventions, generalize beyond bar graphs.

A further interesting venue for prospective research would be to extend our methodology to generally more complex problems. In the present study, we intended to isolate normalization costs and effects of the interventions and therefore used rather simplistic tasks. As our tasks could be solved at a single glance (We intended to obtain high accuracy in order to study response latencies), it would be worth it to study whether the observed effects would also emerge in experiments studying accuracy as the dependent variable or whether they would scale up in displays containing more complex data structures or combinations of multiple graphs (see Moritz et al., 2020).

Finally, our project has established normalization costs for accessing information in data visualizations from non-upright viewing points as well as interventions to reduce these costs on the level of an individual observer. As our research question has been inspired by a collaborative setting in which two (or more) observers access the same depicted data from different viewpoints, an emerging follow-up research question would be on how the observed normalization costs as well as the interventions alter subsequent collaborative processes such as communication (Lyons, 2009) or joint problem solving (Bause et al., 2018).

## Data Availability

Study materials, data, and analyses are available at the Open Science Framework (https://doi.org/10.17605/OSF.IO/YMC35).

## Appendix

See Table

**Table 1:** Detailed descriptive and inferential statistics of the reported experiments

Absolute latencies for response onsets by condition				Test statistics for differences to baseline		
Intervention	Diagram rotation	Label condition	Mean (sd)	*t* (*df*)	*p*	*d*_z_ [95% CI]
Experiment 1						
Letter	Baseline		1574 (283)			
Letter	90	Co-rotate	1693 (344)	1.210 (32)	.235	0.21 [− 0.12, 0.63]
Letter	180	Co-rotate	1750 (420)	2.322 (32)	.027	0.40 [ 0.08, 0.78]
Letter	270	Co-rotate	1737 (380)	2.152 (32)	.039	0.37 [ 0.06, 0.71]
Letter	90	Reformatted	1683 (408)	0.702 (32)	.487	0.12 [− 0.25, 0.45]
Letter	180	Reformatted	1668 (417)	0.360 (32)	.721	0.06 [− 0.31, 0.40]
Letter	270	Reformatted	1679 (389)	0.700 (32)	.489	0.12 [− 0.24, 0.43]
Word	Baseline		1736 (371)			
Word	90	Co-rotate	1904 (408)	6.883 (32)	< .001	1.20 [ 0.85, 1.75]
Word	180	Co-rotate	1917 (433)	5.334 (32)	< .001	0.93 [ 0.63, 1.36]
Word	270	Co-rotate	1904 (414)	6.535 (32)	< .001	1.14 [ 0.76, 1.67]
Word	90	Reformatted	1834 (390)	4.705 (32)	< .001	0.82 [ 0.54, 1.19]
Word	180	Reformatted	1817 (390)	4.533 (32)	< .001	0.79 [ 0.46, 1.23]
Word	270	Reformatted	1793 (358)	3.643 (32)	.001	0.63 [ 0.32, 1.03]
Experiment 2						
Pictograph	Baseline		1496 (425)			
Pictograph	90	Co-rotate	1703 (581)	1.943 (34)	.060	0.33 [− 0.02, 1.00]
Pictograph	180	Co-rotate	1682 (539)	2.029 (34)	.050	0.34 [ 0.00, 0.90]
Pictograph	270	Co-rotate	1698 (570)	1.705 (34)	.097	0.29 [− 0.05, 0.89]
Pictograph	90	Reformatted	1629 (458)	1.169 (34)	.250	0.20 [− 0.12, 0.66]
Pictograph	180	Reformatted	1517 (444)	− 1.124 (34)	.269	− 0.19 [− 0.43, 0.16]
Pictograph	270	Reformatted	1630 (517)	1.036 (34)	.308	0.18 [− 0.15, 0.57]
Word	Baseline		1636 (465)			
Word	90	Co-rotate	1783 (507)	4.189 (34)	< .001	0.71 [ 0.31, 1.38]
Word	180	Co-rotate	1882 (592)	6.007 (34)	< .001	1.02 [ 0.64, 1.63]
Word	270	Co-rotate	1778 (559)	3.120 (34)	.004	0.53 [ 0.10, 1.58]
Word	90	Reformatted	1798 (577)	4.433 (34)	< .001	0.75 [ 0.50, 1.13]
Word	180	Reformatted	1684 (484)	1.826 (34)	.077	0.31 [− 0.04, 1.11]
Word	270	Reformatted	1751 (555)	2.726 (34)	.010	0.46 [ 0.07, 1.32]
Experiment 3						
Multicolor	Baseline		1306 (356)			
Multicolor	90	Co-rotate	1385 (448)	− 0.928 (40)	.359	0.14 [− 0.48, 0.16]
Multicolor	180	Co-rotate	1357 (462)	− 1.607 (40)	.116	− 0.25 [− 0.57, 0.05]
Multicolor	270	Co-rotate	1472 (470)	1.520 (40)	.136	0.24 [− 0.07, 0.60]
Multicolor	90	Reformatted	1397 (469)	− 0.549 (40)	.586	0.09 [− 0.40, 0.22]
Multicolor	180	Reformatted	1331 (399)	− 2.252 (40)	.030	− 0.35 [− 0.70, − 0.05]
Multicolor	270	Reformatted	1416 (450)	− 0.035 (40)	.972	0.01 [− 0.32, 0.31]
Monocolor	Baseline		1526 (469)			
Monocolor	90	Co-rotate	1615 (526)	4.955 (40)	< .001	0.77 [ 0.45, 1.19]
Monocolor	180	Co-rotate	1641 (487)	6.924 (40)	< .001	1.08 [ 0.81, 1.47]
Monocolor	270	Co-rotate	1701 (551)	7.065 (40)	< .001	1.10 [ 0.79, 1.56]
Monocolor	90	Reformatted	1562 (439)	4.925 (40)	< .001	0.77 [ 0.45, 1.18]
Monocolor	180	Reformatted	1607 (499)	5.403 (40)	< .001	0.84 [ 0.55, 1.24]
Monocolor	270	Reformatted	1648 (466)	6.915 (40)	< .001	1.08 [ 0.82, 1.45]

Alpha-level Bonferroni-corrected to .00416

^1^.

## Footnotes

1 Bootstrapped 95% confidence intervals (10,000 repetitions) for all effect sizes are available in the Appendix (Table 1).
